# Tenosynovial Giant-Cell Tumor Presenting as Septic Arthritis of the Knee

**DOI:** 10.5435/JAAOSGlobal-D-20-00089

**Published:** 2021-04-06

**Authors:** Gregory E. Lausé, Michael D. Baird, Kevin P. Krul, Craig R. Bottoni

**Affiliations:** From the Department of Orthopaedic Surgery, Tripler Army Medical Center, Honolulu, HI (Dr. Lausé, Dr. Krul, Dr. Bottoni), and the Department of Orthopaedics, Walter Reed National Military Medical Center, Bethesda, MD (Dr. Baird).

## Abstract

Tenosynovial giant-cell tumor (TGCT) is an intraarticular giant-cell tumor of the synovial tissue and tendon sheaths which often mimics multiple conditions on presentation. This case report describes a previously asymptomatic 67-year-old man with preliminary clinical and laboratory evaluation suggestive of septic arthritis; however, arthroscopy revealed diffuse synovitis, and biopsy confirmed TGCT. To our knowledge, this is the first report of TGCT presenting as septic arthritis in an adult patient. This diagnosis should be considered in evaluation of acute, atraumatic knee pain with associated inflammatory marker elevation.

Tenosynovial giant-cell tumor (TGCT) also known as giant-cell tumor of tendon sheath and pigmented villonodular synovitis is an uncommon proliferative condition of the synovial tissue and tendon sheaths which mimics multiple conditions on presentation and most often meniscal pathology when presenting in the knee. In this case, preliminary clinical and laboratory evaluation suggested an infection; however, arthroscopy and biopsy confirmed TGCT in a previously undiagnosed patient. Despite being uncommon, TGCT should be considered as a potential cause of acute, unilateral joint inflammation without a history of trauma.

## Case Report

**Figure 1:**
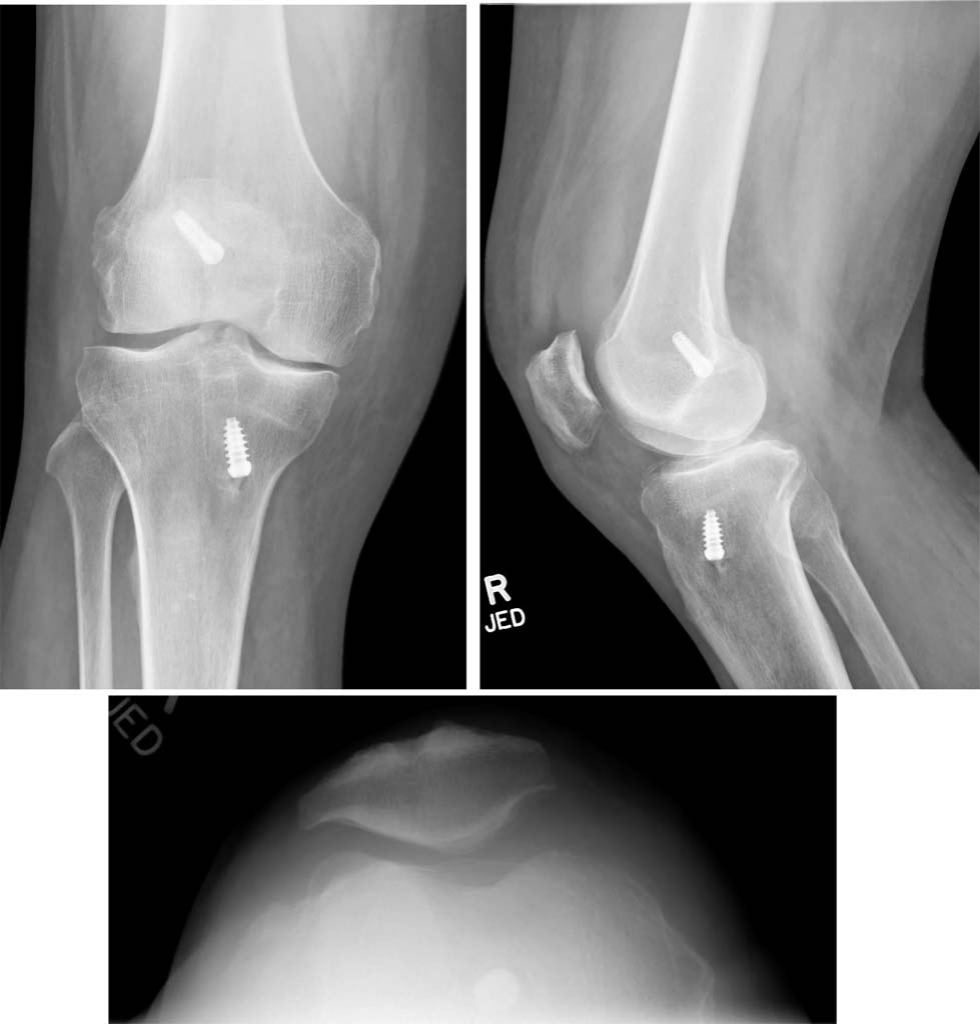
Radiograph showing standard Anterior-Posterior (AP), lateral and sunrise patella views of the knee showing earlier ACL reconstruction, moderate-to-severe tricompartmental osteoarthritis, and moderate joint effusion.

A 67-year-old man presented to the emergency department with a 3-day history of progressive right knee pain and inability to bear weight on the affected leg. In the emergency department, he was afebrile and had no reported history of trauma. Plain radiographs of the knee demonstrated evidence of an effusion with moderate-to-severe osteoarthritis and intact screws from a 1996 anterior cruciate ligament (ACL) reconstruction (Figure 1). An arthrocentesis was done and showed a cell count of 43,000/mm^3^ white blood cells with 95% leukocytes and no crystals. Gram stain showed 3+ polymorphonuclear neutrophils (PMNs) and 1+ monocytes. Erythrocyte sedimentation rate and C-reactive protein were both elevated to 16 mm/hr and 6.09 mg/dL, respectively. The remainder of his laboratory evaluation to include complete blood count (CBC), BMP, and uric acid were all within normal limits.

**Figure 2:**
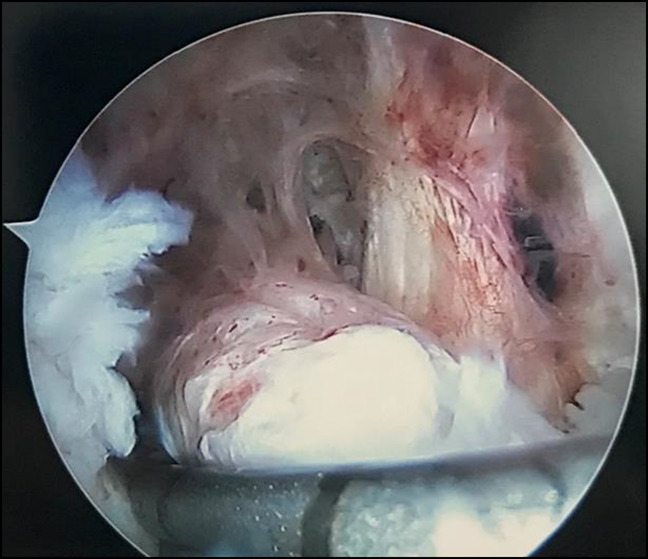
Image showing arthroscopic view of notable synovitis and synovial mass of the medial aspect of the suprapatellar pouch.

**Figure 3:**
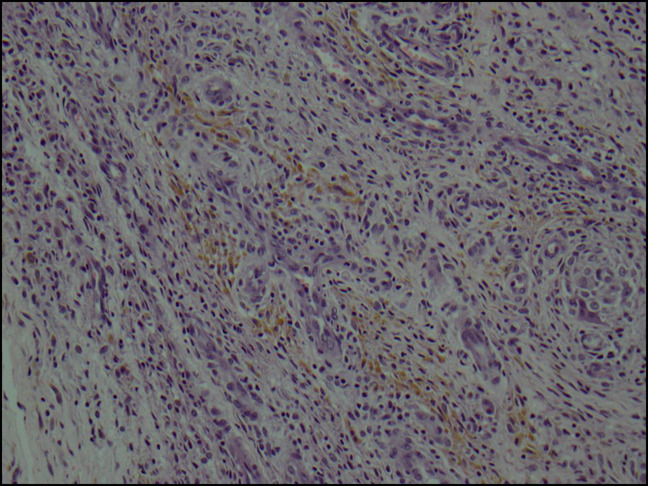
Microscope image of the tissue specimen from our patient’s knee showing hemosiderin deposition consistent with TGCT.

The patient was admitted and taken to the operating room for arthroscopic irrigation and débridement of the right knee for presumed septic arthritis based on his clinical presentation. Magnetic resonance imaging (MRI) is not part of our standard workup for infection and was not obtained before surgical intervention. Cultures were taken intraoperatively, and diagnostic arthroscopy revealed notable synovitis throughout the knee and a mass invested in the synovium (Figure 2). There was also evidence of articular erosion in the medial compartment. The mass was excised and sent for biopsy, later confirmed as TGCT (Figure 3). The patient was discharged after three sets of cultures remained negative and had down trending inflammatory markers. At the 2-week, 6-month, and 2-year follow-up, the patient demonstrated no evidence of recurrent symptomatic pathology.

## Discussion

In general, TGCT is uncommon, with the reported incidence typically quoted at 1.8 cases per million people.^1^ Among these cases, the knee is believed to be affected in two of every three patients.^2^ It is classically described as either diffuse, where multiple areas of the synovial lining are involved, or localized, where there is typically a single-pedunculated lesion. In 2013, the World Health Organization reclassified localized (LTGCT) to include giant-cell tumor of tendon sheath and nodular tenosynovitis, whereas diffuse (DTGCT) encompasses diffuse-type giant-cell tumor and pigmented villonodular synovitis.^3^ Although TGCT may occur at any age, LTGCT is commonly present in the fourth and fifth decades of life, whereas DTGCT is present earlier (<40 years).^4^ Variation exists in describing which subtype is more common, but one study reported that LTGCT is seven times as common as DTGCT.^5^ The reclassification emphasizes that the driving force is a tumor and the symptoms are secondary to an inflammatory response within the joint. There is a genetic relationship in both LTGCT and DTGCT seen with chromosomal translocation-t (1; 2) (CSF1; COL6A3) at 1p11-13, a site for the Colony Stimulating Factor 1 (CSF-1) gene and other changes that result in overexpression of CSF1R.^6^ These neoplastic cells recruit macrophages bearing the CFS1R receptor, differentiate into multinuclear cells, and create the aggressive multinuclear inflammatory cells including giant cells, macrophages, and osteoclasts.^7^

The presentation of TGCT can vary both among subtype and regarding the variety and duration of symptoms. Patients with LTGCT may be symptomatic for years and not seek medical attention.^5,8,9^ The presentation commonly includes pain, swelling, decreased activity, and mechanical symptoms, and physical examination findings consistent with meniscal pathology.^10,11^ In one study examining patients with LTGCT, the authors found that almost all reported pain on presentation, whereas over half presented with an effusion.^5^ Presentation with symptoms similar to acute infection is exceedingly rare and, as such, is not included in the differential diagnosis for TGCT. Only one other case report from Hong and Hing^12^ described a 10-year-old boy who had knee swelling and a presentation highly suggestive of an infectious arthritis.

Our case represents an atypical presentation of TGCT presenting as acute septic arthritis. The patient did not describe any history of trauma to the affected joint. Furthermore, he noted only a few days of symptoms and swelling, a sharp contrast to the literature describing about 2 to 5 years of symptoms before patients commonly present. With a leukocyte count of 43,000 cells/mm^3^ with 95% PMNs, elevated inflammatory markers and the acuity of his presentation, infection, or crystallopathy were the immediate concerns. Although his inflammatory markers were not markedly elevated, perhaps owing to the inflammatory process of TGCT, rather than infectious, the mild elevation can still be consistent with septic arthritis.^13^ Therefore, our patient was consented for an arthroscopic irrigation and débridement and started on empiric antibiotics after aspiration. All preoperative and intraoperative cultures taken prior to antibiotic administration were negative.

When diagnosing TGCT, plain radiographs are frequently normal and thus provide limited utility.^5,14^ MRI shows a characteristic soft-tissue mass with focal hypointense areas on T1 and T2 and has been shown to be sensitive, but not specific.^8,15^ Associated joint effusion may be described on MRI, particularly in large joints. In addition, gradient echo images show enlarged areas of decreased signal intensity which has been described as “blooming” by Murphey et al.^15^ “Blooming” is secondary to hemosiderin-laden tissue and is strongly correlated with TGCT and nearly diagnostic on MRI. Diagnosis is best made on arthroscopy and can be confirmed histologically.^11^ Microscopically, TGCT is described as polyhedral synovial-like cells with interspersed multinucleated giant cells and foam cells with hemosiderin deposition in areas of hyalinized collagen tissue.^5^ In addition, the observance of foam cells has caused some to speculate that TGCT is also involved with a disturbance in lipid metabolism.^16^

Excision of the lesion is the best treatment.^17^ In one review, 40 patients with unilateral DTGCT and all 40 had recurrence after arthroscopy.^10^ Another study found that the rate of recurrence was 21.7% in patients with DTGCT but that it was one-third of that in patients with LTGCT. Two reviews comparing arthroscopic and open synovectomy found no difference in recurrence, postoperative complications, or osteoarthritis incidence.^18,19^ However, Auregan et al recommended against incomplete synovectomy for patients with DTGCT type because of the high risk of recurrence, whereas other studies strongly recommend radiation therapy to reduce recurrence in diffuse pigmented villonodular synovitis (DPVNS).^20,21^

In contrast to patients with DTGCT, patients with LTGCT have been shown to have a high rate of cure with local excision. The Dines et al^5^ study, which assessed a subgroup of patients with LTGCT only, found that all of their patients had full range of motion with no pain or swelling on the follow-up, which was over 18 months after treatment on average and 90% of their patients described “excellent results” 5 years after treatment.

## Conclusion

This case illustrates that TGCT should be considered as part of the differential for a patient with acute onset knee pain and effusion without a history of trauma and with a lack of systemic symptoms. Arthroscopic treatment in these patients should be highly considered because it is both diagnostic and therapeutic in patients with localized disease.
